# From Wellbeing to Social Media and Back: A Multi-Method Approach to Assessing the Bi-Directional Relationship Between Wellbeing and Social Media Use

**DOI:** 10.3389/fpsyg.2021.789302

**Published:** 2021-12-24

**Authors:** Nastasia Griffioen, Anna Lichtwarck-Aschoff, Marieke van Rooij, Isabela Granic

**Affiliations:** ^1^Games for Emotional and Mental Health Lab, Behavioural Science Institute, Department of Developmental Psychopathology, Radboud University Nijmegen, Nijmegen, Netherlands; ^2^Faculty of Behavioural and Social Sciences, University of Groningen, Groningen, Netherlands; ^3^Faculty of Behavioural, Management and Social Sciences, University of Twente, Enschede, Netherlands

**Keywords:** social media, affective wellbeing, stress, stimulated recall, user-centric methods, emerging adults, stress regulation

## Abstract

Literature concerning the relationship between social media use and wellbeing is inconsistent in its findings, and most research has focused on time spent on social media rather than on what emerging adults do there, with whom and why. Here, we investigated whether momentary social stress affects emerging adults’ social media use, and whether this social media use relates to subsequent changes in wellbeing. We implemented a multi-method paradigm utilising objective and self-report data to investigate how social stress relates to how (much) and why emerging adults use social media. We report on findings based on 114 17–25-year-old emerging adults recruited on university campus. Our findings suggest that social stress does not affect adolescents’ subsequent social media use and that there is no relationship between social media use after stress and changes in momentary wellbeing. Our work illustrates the need for detailed approaches in social media and psychological wellbeing research.

## Introduction

In recent years, youth wellbeing has seen an alarming decrease, with over one in five adolescents reporting feelings of stress, anxiety and depression (Office for National Statistics, 2017). Simultaneously, digital forms of communication, and especially social media, are booming in popularity. In the last few years, the percentage of social media users in the US has increased to 65% for the entire population, and to 90% for 18–29-year-olds (Pew Research Center, 2015). The co-occurrence of these two trends has sparked the suggestion by some that the wellbeing and social media trends may in fact be related and that social media may contribute to the decrease in mental health among youth (Tromholt, 2016; Twenge, 2017; Verduyn et al., 2017; Barr, 2019).

Although much work has already been directed at social media and its effects on wellbeing, the field is still characterised by inconsistent findings and methodological shortcomings (Orben et al., 2019; Orben, 2020). Moreover, little attention has been given to the possibility that youth’s social media use might be affected by their momentary affective wellbeing, for instance when coping with stress, thus reversing the causal arrow. Here, using a novel user-centric approach, we aimed to determine whether changes in wellbeing due to momentary social stress lead to differences in social media use, and whether subsequent social media use relates to improvements in momentary wellbeing.

### Importance of Detailed Approaches to Social Media Use

Social media use has been linked extensively to both decreases and increases in wellbeing (Levenson et al., 2016; Kim, 2017; Hardy and Castonguay, 2018; Riehm et al., 2019). Particular focus has been placed on the potential negative consequences of social media use. ‘Social media fatigue’ or stress—feelings of tiredness and being overwhelmed by social media’s pull—has been well-documented by researchers over the past couple of years (Bright et al., 2015; Maier et al., 2015; Beyens et al., 2016; Dhir et al., 2018, 2019). Fox and Moreland (2015) for instance found that adults in their study reported feeling stressed as a result of Facebook use due to a lack of privacy, having to manage content they did not wish to see and its potential for relational tension and conflicts.

Reviews and meta-analyses, however, indicate that the field is far from conclusive and that the relationship between social media use and wellbeing is not simple. In fact, which form this relationship takes may depend on the specifics of social media use (Burke et al., 2010b) rather than the global indices—such as time spent on social media—that have so far been at the centre of attention (Best et al., 2014; Orben et al., 2019; Orben, 2020). Recently, what youth do exactly on social media has started to receive increased attention (Burke and Develin, 2016; Eschler et al., 2020; Huang et al., 2020; Trieu and Baym, 2020), but detailed data on social media use specifics are still scarce.

The methods that have been employed to study the relationship between social media use and wellbeing may play a large part in explaining why results have been so inconclusive so far. In a recent review, we have shown that most studies use retrospective questionnaires to assess social media use (Griffioen et al., 2020b), which have been shown to poorly reflect actual media use (Ellis et al., 2019; Ernala et al., 2020). Additionally, many studies are correlational in nature (Orben, 2020). This is problematic, given that wellbeing might in fact influence to what extent and in what ways social media are interacted with, rather than (only) vice versa.

### Social Media Use as Stress Relief

In fact, multiple studies report that adolescents and young adults use social media as a way of dealing with stress and to improve their wellbeing (Van Ingen and Wright, 2016; Rus and Tiemensma, 2017). Social media have indeed been shown to benefit users in a number of ways. For instance, social media have been found to have the potential to contribute to users’ social capital: the resources and advantages people enjoy through their relationships with other people (Ellison et al., 2007; Steinfield et al., 2008; Valenzuela et al., 2009; Chen and Li, 2017; Pang, 2017). Furthermore, there are findings suggesting that social media are used to give and receive social support (DeAndrea et al., 2012; De Choudhury and De, 2014). Especially in times of stress, when wellbeing is under pressure, the social affordances offered by social media may facilitate stress and wellbeing regulation. Connections with others allow us to more adequately cope with stressors (Beckes and Coan, 2011), and it is often through these connections that we form and try to maintain our self-worth and self-esteem. This re-affirmation of our values has been shown to play an important role in coping with stressors (Creswell et al., 2005; Sherman et al., 2009; Brady et al., 2016).

Additionally, there is growing empirical evidence that re-affirmation of one’s self-value after ego threat can be a driving force for social media use (Toma and Hancock, 2013). Social media present an excellent environment for self-affirmation because they offer overviews and representations of a number of aspects that are crucial to users’ self-definition: social ties and roles, interests, affiliations, values and the extent to which they are connected to the social environment around them. Toma and Hancock (2013) indeed found that, after an ego threat, participants had a tendency to visit their social media profiles, most likely in an attempt to repair the damage to their self-worth through re-affirmation of the self. A recent study by Coates and colleagues (Coates et al., 2019) similarly suggests that social media use might be successful at coping with and reducing stress, either through the opportunities for social connection and support that they afford, or through providing a way to momentarily escape the stressor(s) at hand. There are thus clear empirical indications that the bi-directional relationship between wellbeing and social media use is an avenue worth exploring.

It seems likely that stress (in particular social stress) might affect the ways in which social media are used and that social media use may indeed be related to improvements in momentary wellbeing following stress. It is therefore important to consider which aspects of social media use to focus on. First, recent studies have indicated that whether social media are used actively vs. passively may play an important role in understanding the relationship between social media use and wellbeing (Burke et al., 2010a,b; Frison and Eggermont, 2016). In particular, passive social media use has often been associated with decreased indices of wellbeing (Shaw et al., 2015; Verduyn et al., 2015, 2017), whereas a more active approach to social media seems to be related to increased wellbeing (Deters and Mehl, 2013; Frison and Eggermont, 2015; Shakya and Christakis, 2017). Second, the social ties that youth come into contact with on social media seem to matter to how youth experience their social media use. Positive interactions on social media seem to stem primarily from close social ties, such as family, friends and romantic partners (Bayer et al., 2016), and being in touch with close social ties (i.e., friends, family and romantic partners) has been shown to be related to better mental health outcomes (Rae and Lonborg, 2015). Last, emerging adults’ motivation for using social media may also help shed light on the circumstances in which social media are related to better or worse wellbeing states. For instance, it is important to consider whether social media are used out of boredom or with a specific goal in mind (Manuoğlu and Uysal, 2019): in line with self-determination theory (Deci and Ryan, 1980, 2004), Manuoğlu and Uysal (2019) found that self-determined, intrinsic motivations for social media activities were related to increased wellbeing, whereas extrinsic motivations for social media use were related to decreased wellbeing.

These specific aspects of social media use may play different roles in disentangling relationships between social media use and regulation of wellbeing. Additionally, we do not know of any studies that have assessed how youth feel in the moment, when using social media in particular ways. In order to achieve the necessary sensitivity to context and function of social media use, a new approach is needed.

### Current Study

The existing body of work has shown that (young) people’s interaction with social media is a complex phenomenon that needs to be investigated not only with more methodological rigour, but also with more attention to why, how and with whom social media are used. Therefore, in the current study, we have implemented a novel paradigm called ‘stimulated recall of social media use’ (Griffioen et al., 2020a). This paradigm entails a combination of detailed and objective data with an intensive, semi-structured interview and allows researchers to delve deep into people’s personal experiences when interacting with digital technologies like social media, without compromising the reliability of data. This paradigm was developed specifically to reliably assess social media use, and to provide researchers with a deeper, context-sensitive understanding of how and why youth use social media.

We assessed whether stress affects social media use both in terms of time spent on social media, as well as the types of activities youth engaged in on social media, and the social ties and motivations associated with this social media use. (H1) First, we hypothesised a lack of stress’ effect on quantity of social media use, given recent literature suggesting that time spent on social media is not an informative metric when it comes to psychological wellbeing effects (Coyne et al., 2020; Granic et al., 2020). (H2) Second, we hypothesised an effect of the stressor on the level of activity displayed while using social media, either leading to more or less active use than in the control condition. Although this lack of directionality seems problematic, this is simply because multiple different mechanisms may underlie this hypothesised effect. On the one hand, young people might engage more actively with social media in an attempt to feel more connected to their social network and thus upregulate their wellbeing, as has been illustrated by research demonstrating a tend-and-befriend response to stress (Taylor et al., 2000; von Dawans et al., 2012). On the other hand, withdrawal from social engagement in the face of stress is also possible and has been found to be used by adolescents as means for coping with stress, especially in situations marked by stress related to the self (Seiffge-Krenke et al., 2009), and a lack of control (Ebata and Moos, 1994). No hypotheses were formed for the effect of the stressor on young people’s motivations for using social media, or proportion of close-tie interactions when using social media.

Additionally, we investigated whether post-stress social media use was related to changes in our indices of momentary wellbeing, and thus to recovery after a stressor. We also assessed whether such a relationship might depend on the specifics of social media use, such as the motivation, activity and social ties involved. We again hypothesised that time on social media would not play a significant role (H3), whereas activity of social media use, motivations and social ties involved would. In line with the existing literature discussed above, we expected that more active social media use (H4), more goal-directed (and thus self-determined) motivations (H5) and more close-tie interaction when using social media (H6) would be related to greater improvement on our indices of wellbeing. For a more comprehensive overview of our hypotheses, see our preregistration.^1^ For all research questions, stress symptoms during the past week were included as a moderator to explore the role of ‘baseline’ levels of stress in the interplay between changes in wellbeing and social media use.

## Materials and Methods

### Participants

A total of 125 participants took part in this study after having signed up *via* the university’s online participant registration tool. Eligibility requirements for participation consisted of being between 18 and 25 years of age and being a native Dutch speaker. Three participants turned out not to be native Dutch speakers. Their test session was conducted in English. The study on average took 1.5 h per participant to complete, and participants were compensated with either €15 gift cards or 1.5 study participation credits. A number of participants (*n* = 11) had to be excluded from analyses, either because they had not consented to the use of video footage (*n* = 4) or aborted the experiment earlier on (*n* = 1), or because the video footage turned out to be of insufficient quality for a reliable stimulated recall interview (*n* = 6). Most participants were female (*n* = 99) and their average age was 21.22 (*SD* = 2.17).

### Measures

**Figure 1:**
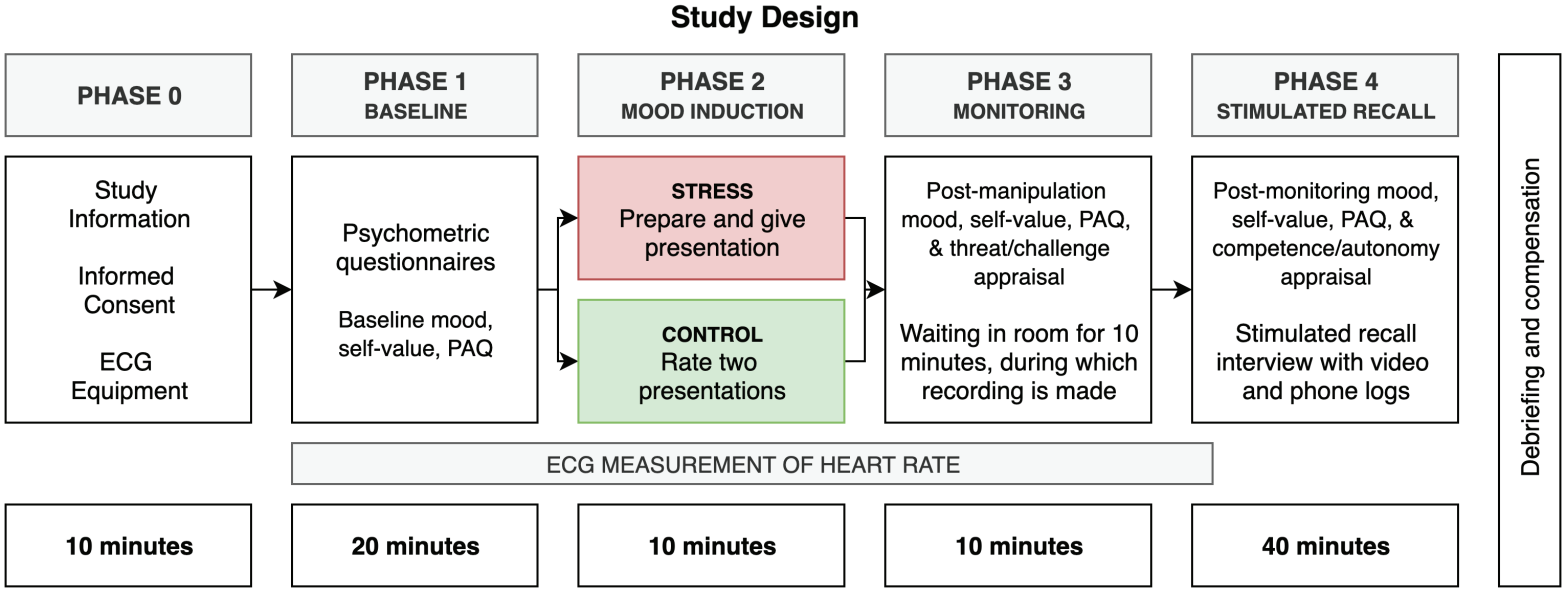
Schematic representation of the study design.

All information from participants’ stimulated recall charts was transferred to a dataset. All social media measures were thus computed over the 10-min monitoring phase. Bursts are the ‘general actions’ that are denoted in the first row of the stimulated recall chart (see Figure 1), whereas actions are the specific behaviours done within such a burst as denoted in the third row of the stimulated recall chart.

#### Time Spent on Social Media

Time spent on social media was calculated by adding up the durations (in seconds) of all social media bursts. Social media activity was defined as the use of the following apps: Facebook, Twitter, Tinder, Tumblr, TikTok, Instagram, Snapchat, Reddit, LinkedIn, YouTube, Quora, Jodel and Polarsteps.

#### Proportion of Active Social Media Actions

Proportion of active social media actions was calculated by dividing the number of active social media actions (i.e., posting, commenting, liking and sharing) by the total amount of social media actions.

#### Proportion of Close-Tie Social Media Actions

Proportion of close-tie social media actions was calculated by dividing the number of times participants linked a close-tie connection (i.e., friend, family and romantic partner) to social media actions, by the times a distant-tie connection (i.e., acquaintance, stranger and celebrity) was linked to actions.

#### Proportion of Goal-Directed Social Media Bursts

Proportion of goal-directed social media instances was calculated by dividing the number of times a goal-directed motivation was linked to social media instances, by the total amount of social media use bursts.

#### Change in Momentary Wellbeing

**Figure 2:**
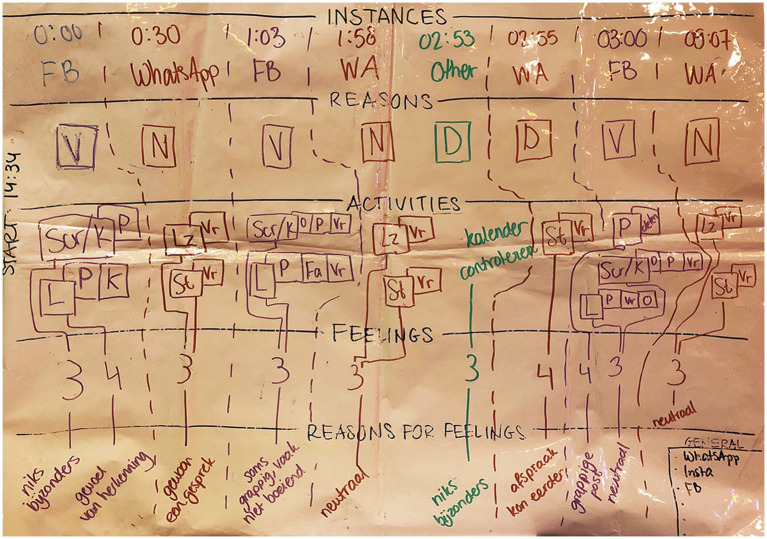
An example of a filled-out stimulated recall chart.

Change in momentary wellbeing during monitoring phase was operationalised as the difference in mood, self-value, heart rate and subjective physiological arousal (PAQ) score between the post-stress and post-monitoring measurements (respectively, at the start of Phase 3 and Phase 4, see Figure 2). Mood and self-value were measured using a 5-point smiley scale (ranging from very negative to very positive), where participants were asked to answer, ‘How would you rate your mood in this moment?’ (mood), and ‘How do you feel about yourself in this moment?’ (self-value). Heart rate data were collected using the BITalino (r)Evolution Plugged (Batista et al., 2019). Electrodes were applied to the participants’ chest in accordance with the 3-electrode ECG placement (Drew et al., 2004). Subjective physiological arousal was measured using the Physiological Arousal Questionnaire (PAQ; Dieleman et al., 2010).

#### Pre-existing Stress

Participants’ baseline (i.e., pre-existing) stress score was computed as the total sum score of all Stress items of the Depression, Anxiety and Stress Scale (DASS-21; Lovibond and Lovibond, 1995; De Beurs, 2010).

### Procedure

Figure 2 provides a schematic overview of the study procedure. Participants were tested in the Behavioural Science Institute’s ‘bar lab’ (Bot et al., 2005) at Radboud University Nijmegen, which was chosen for its informal and inviting atmosphere, aimed at eliciting natural behaviours in participants. Before coming into the bar lab, participants were assigned to one of two conditions using simple randomisation (Control vs. Stress). Upon coming into the lab, participants were asked to read the study information letter and sign an informed consent form. ECG measurement equipment was applied to the participants’ chest. Subsequently, participants were asked to turn their phones off and on, supposedly to perform BITalino calibration checks. In reality, this was done to (1) check whether participants had brought their phones and (2) to nudge participants to have their phone close-by for the monitoring phase.

After the physiological equipment was set up, participants were asked to fill out psychological questionnaires as well as baseline measurements of their mood, self-value and subjective physiological arousal (see Figure 2, Phase 1).

Participants assigned to the Control condition were then asked to perform a neutral task, which consisted of rating two 5-min presentations on video using a checklist provided by the experimenter. Participants in the Stress condition were instructed to perform the Leiden Public Speaking Task (LPST; Westenberg et al., 2009). The LPST has been shown to elicit a moderate stress response in young adults. Tasks, such as the LPST, have been used extensively in psychological research to examine the effects of induced stress on subsequent tasks (Payne et al., 2002; Roelofs et al., 2005; Guez et al., 2016). The LPST consists of participants being given a short amount of time (e.g., 5 min) to prepare a presentation, after which they are asked to present in front of a pre-recorded audience while being recorded.

Following the manipulation phase, both groups were asked to fill out the post-stress measurements (see Figure 2, Phase 3). Participants were then asked to wait in the room for 10 min while the experimenter went to ‘help a colleague with another participant’. This marked the start of the ‘monitoring phase’, during which participants had been instructed to remain seated at the table and ‘not disturb the physiological measurement’. They were also told that they could do whatever they wanted while the experimenter was helping out their colleague. If the participant had a bag with them, the bag was placed next to the participant so that they would be able to retrieve anything from there that they would need or like to use. If participants inquired whether it would be O.K. for them to use their phone, the experimenter would indicate that it was fine, since calibration already had confirmed that there was no interference with the physiological signal. After any questions from the participant had been answered, the experimenter reiterated that they would be back in about 10 min after helping their colleague set up.

Upon leaving the bar lab, the experimenter quickly moved to an adjacent control room, where an overhead video camera was switched on to record participants’ activities in the bar lab during the 10-min monitoring phase. This camera was positioned in such a way that the participant was not identifiable, and no text or images could be read on the participant’s phone (Griffioen et al., 2020a). Only the participant’s hand movements (e.g., clicking, typing and swiping) and the general colour and layout of what was on the screen could be made out. Upon the experimenter’s return, participants were thanked for their patience and asked to fill out the post-monitoring questions (see Figure 2, Phase 4).

Next, participants were partially debriefed and told that the true aim of the study was to gain insight into what youth do on their phones in their spare time. The debrief was partial, because at this point the participants were not yet told that we were interested in the potential effect of the stressor they had just experienced (in those cases that the participant had been assigned to the Stress condition). The physiological recording was stopped at that point. Participants were told that the experimenter would like to spend the remainder of the study time on an interview with the participant to discuss these past 10 min and that a video recording had been made to aid the participant in their recollection of the events in this time [for a more detailed description of the paradigm, see (Griffioen et al., 2020a)]. If a participant did not consent to the use of the video footage for the interview (*n* = 4), the interview did not take place, and the participant was fully debriefed and given a reward corresponding to the time spent in the study.

After the participant had provided informed consent for the use of the video recording, we proceeded with the stimulated recall interview during which the experimenter assessed participants’ activities, durations of these activities and the associated motivations and feelings during the 10-min waiting period, which were mapped out on a ‘stimulated recall chart’ (see Figure 1). Participants were explicitly invited to actively participate in this act of ‘co-research’, where the participant and experimenter together tried to uncover the intricacies and functions of their smartphone use. This also ensured that the act of being recorded and subsequently having to view that recording with a stranger felt less privacy intrusive.

The stimulated recall interview started off by asking participants to recall what they had been doing during the monitoring period without the help of video or phone data. Then, the interview commenced by the experimenter starting playback of the video footage and asking the participant what they were doing at a given time point, recording the video time stamp corresponding to the start of a new activity and prompting the participant to talk about why they had started to engage in the activity, what exactly they were doing and how they felt while engaging in that activity. In places where the video footage did not provide sufficient detail for accurate recollection, participants were asked whether they would be willing to look up information about what they had done on their phones (e.g., in social media data logs or messaging logs). The entire video footage of 10 min would be processed and discussed in abovementioned fashion, a process that on average took about 30–45 min.

After the interview, participants were thanked for their participation and were asked to fill out a final debrief questionnaire to check whether they knew—at the time of the monitoring phase—that we were interested in their phone use. The study protocol was approved by the Ethics Committee of the Faculty of Social Sciences at Radboud University Nijmegen (approval number ECSW-2019-020).

### Analyses

Data of 114 participants (Control condition *n* = 59; Stress condition *n* = 55) were analysed. Hypotheses and analyses were preregistered after data collection but prior to analyses (see footnote 1). Small deviations from the preregistered analyses have taken place, documented in a List of Explanations and Deviations from Preregistration on OSF.^2^ All analyses have been done in R Studio using R version 3.6.2 (RStudio Team, 2019). ANOVAs and multiple regressions were conducted to answer our research questions.

## Results

Descriptive statistics on all variables of interest in this study can be found in Table 1.

**Table 1:** Descriptive statistics.

Variable	Control condition				Stress condition			
	Mean ± SD	Median (IQR)	Min.	Max.	Mean ± SD	Median (IQR)	Min.	Max.
Time on social media (in seconds)	225.59 ± 198.41	184 (24, 405)	0	600	226.13 ± 210.01	134 (24, 420)	0	600
Prop. active social media actions	0.25 ± 0.23	0.27 (0, 0.48)	0	0.67	0.19 ± 0.23	0 (0, 0.40)	0	0.67
Prop. close-tie social media actions	0.33 ± 0.23	0.37 (0.19, 0.50)	0	1	0.25 ± 0.29	0.20 (0, 0.37)	0	1
Prop. goal-directed social media bursts	0.43 ± 0.26	0.40 (0.29, 0.59)	0	1	0.41 ± 0.31	0.40 (0.17,0.60)	0	1
Change in mood during monitoring	0 ± 0.50	0 (0, 0)	−1	1	0.27 ± 0.62	0 (0, 1)	−1	2
Change in self-value during monitoring	0 ± 0.46	0 (0, 0)	−1	1	0.25 ± 0.64	0 (0,1)	−1	2
Change in heart rate during monitoring	−1.82 ± 3.76	−1.04 (−3.48, 0.27)	−12.87	5.66	−9.50 ± 5.43	−8.99 (−12.12, −6.12)	−30.94	4.80
Change in subjective arousal during monitoring	−1.84 ± 3.77	−2 (−4, 0)	−10	7	−8.45 ± 6.82	−8 (−11.50, −3)	−32	3
Stress score	8.31 ± 7.16	6 (2, 12)	0	26	8.80 ± 6.37	8 (4, 14)	0	22

*SD, standard deviation; IQR: interquartile range*.

### Effect of Stress on Wellbeing and Social Media Use

We found no significant main effects of condition (Stress vs. Control) on post-manipulation mood [*F*(1,109) = 2.61, *p* = 0.109] or self-value [F(1,109) = 3.29, *p* = 0.073]. To determine whether stress levels prior to coming into the lab may have interacted with the stress manipulation, stress score was included as a moderator in the manipulation check. For mood, an interaction effect was found [F(1,109) = 7.87, *p* = 0.006]: post-manipulation mood levels were lower in the Stress condition than in the Control condition (as hypothesised), but only for participants with low levels of pre-existing stress; for high stress scores, no difference in post-manipulation mood between conditions was found. This suggests that, for these participants, higher pre-existing levels of stress seem to buffer against effects of acute stress on mood, and that only people who were fairly stress-free to begin with were affected in their mood by the stressful task. No interaction effect with stress scores was found for post-manipulation self-value. Thus, the manipulation did not have the desired effect on post-manipulation self-value, and only had the desired effect on post-manipulation mood for participants who were low on stress to begin with.

Furthermore, we found significant effects of the stress manipulation on post-manipulation subjective physiological arousal [*F*(1,108) = 28.47, *p* < 0.001, Stress > Control] and average manipulation phase heart rate [*F*(1,103) = 15.03, *p* < 0.001, Stress > Control]. For post-manipulation subjective physiological arousal scores, no interaction effect with stress scores was found. However, we found an interaction of average manipulation phase heart rate with stress scores: similarly to the interaction effect on post-manipulation mood, the effect of the manipulation on heart rate was present at low levels of pre-existing stress (Stress > Control) but seemed to dissolve when stress scores were high [*F*(1,103) = 4.61, *p* = 0.034]. In other words, the manipulation did have the desired effect on both subjective physiological arousal and heart rate, but again, mostly for participants with low stress scores.

Looking at the effects of stress on social media use, we found no effect of the stress manipulation on the amount of time (in seconds) spent on social media during our 10 min monitoring phase [*F*(1,110) = 0.00, *p* = 0.989]. We also found no effect of the stress manipulation on the proportion of active social media actions [*F*(1,86) = 1.16, *p* = 0.284], nor on the proportion of social media actions that were related to close social ties [*F*(1,86) = 2.43, *p* = 0.123], nor on the proportion of goal-initiated social media actions [*F*(1,86) = 0.90, *p* = 0.346]. No interactions were found with stress score. In sum, stress did not seem to influence any aspect of social media use shortly after the stressor.

### Social Media Use and Wellbeing Recovery After Stress

Since mood and self-value were not affected by the stress manipulation, analyses regarding recovery after stress do not include mood and self-value [also see our List of Explanations and Deviations from Preregistration (see footnote 2)].

#### Quantity of Social Media Use

The overall regression model of time spent on social media as a predictor of change in average heart rate during the monitoring phase [*F*(3,47) = 3.03, *p* = 0.039, *R*^2^ *=* 0.16] was significant. Within this model, ‘time spent’ was itself not a significant predictor, meaning that the amount of time participants spent on social media was not related to changes in heart rate during the monitoring phase. However, within the overall model, stress score was a significant predictor (*b* = 0.47, *p* = 0.007), indicating that a higher stress score was related to a slightly smaller decrease in heart rate in the period after the manipulation phase.

**Figure 3:**
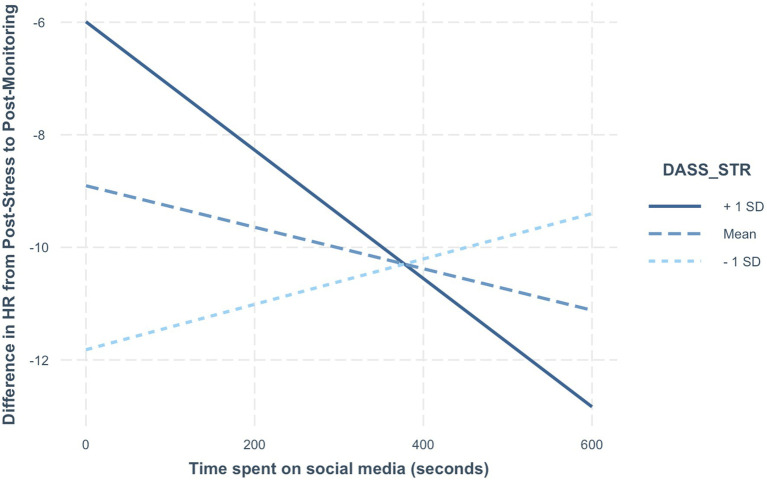
Interaction between Time on Social Media and Pre-existing Stress score.

Additionally, the interaction term between time spent on social media and stress score proved also to be a significant predictor of changes in heart rate during the monitoring phase (*b* = −0.001, *p* = 0.020), such that more time spent on social media was related to a greater decrease in heart rate during the monitoring phase (i.e., greater recovery), but only for participants scoring high on pre-existing stress (see Figure 3). Specifically, the slope of time spent on social media starts being significant for stress values higher than 12.96, as pointed out by a Johnson-Neyman interval calculation. In contrast, the overall regression model of time spent on social media as a predictor of change in subjective physiological arousal score during the monitoring phase however was not significant [*F*(3,47) = 0.31, *p* = 0.817, *R*^2^ *=* 0.02]. In other words, more social media use was only related to greater stress recovery in terms of heart rate, and only for participants scoring ‘high’ on pre-existing stress, which is operationalised by us as being above Lovibond & Lovibond’s cut-off of 14, indicating the upper bound of the ‘normal’ stress levels category. The majority of participants scored within the normal (0–14) range (*n* = 94), and the rest scored in one of the higher categories of stress (*n* = 20).

#### Qualities of Social Media Use

The overall regression model of proportion of active social media behaviours, proportion of close-tie social media behaviours and proportion of goal-initiated social media behaviours and their interactions with stress scores as predictors of change in average heart rate during the monitoring phase was non-significant [*F*(7,32) = 1.35, *p = 0*.259, *R*^2^ *=* 0.23]. Similarly, the overall regression model containing the same independent variables and their interactions with stress scores as predictors of change in subjective physiological arousal score during the monitoring phase was also non-significant [*F*(7,32) = 1.91, *p* = 0.100, *R*^2^ = 0.30]. In sum, how or why social media were used after a stressor did not relate to subsequent recovery of heart rate or subjective physiological arousal.

## Discussion

In the present study, we used a novel, user-centric research method to investigate the specifics and functions of young adults’ interactions with social media. Specifically, we investigated the bi-directional relationship between social media use and four different aspects of momentary wellbeing: mood, self-value, heart rate and subjective physiological arousal. First, we assessed whether momentary social stress (i.e., having to prepare and give an unexpected presentation) affected subsequent social media use, both in terms of time spent on social media, as well as specific aspects of social media use. In line with our first hypothesis, momentary social stress did not affect how much time participants spent on social media in the 10 min following the stressor. Additionally, and contrary to our second hypothesis, social stress also did not cause any differences in the qualitative aspects of social media use (i.e., why and how social media were used, as well as which social ties were involved).

Second, we assessed whether social media use after the stressor was related to changes in mood, self-value, subjective physiological arousal and heart rate. Again, contrary to our expectations outlined in our fourth, fifth and sixth hypotheses, we found no direct relationships between characteristics of social media use (qualitative or quantitative) and our indices of momentary wellbeing. Furthermore, contrary to our third hypothesis, we did find that more time spent on social media was related to greater decreases in heart rate (and thus, a greater recovery), but only for participants who came into the lab with relatively high stress levels in the past week (see Figure 3). Although we want to point out that this was an unexpected and potentially incidental finding, it may suggest that for people who are already stressed, spending more time on social media in the 10 min following a new stressor is related to greater physiological recovery, and is in that way beneficial.

In contrast to what we had hypothesized and also in contrast to earlier studies, our research suggests that there is no relation between stress and social media, and vice versa. Given the more objective nature of our data compared to earlier studies, this finding’s deviation from what we know based on earlier studies may simply be due to increased validity and specificity of the data used in the present study. It remains important, however, to consider a number of methodological aspects to the present study in view of the found lack of associations.

First, while the lack of the stress condition’s effect on mood and self-value may at first glance look like a failed manipulation, we maintain that this is not the case. A review of stress inductions and their relation to subjective measurements of their effects (such as on mood and self-value) has found that more often than not (i.e., in approximately 75% of the studies) a correspondence between objective (physiological) measures and subjective (psychological) measures of stress is lacking (Campbell and Ehlert, 2012). Although this discrepancy (i.e., finding an effect on objective measures but not on subjective measures) may seem problematic for one’s ability to conclude that participants experienced stress, relatively ‘harmless’ reasons for this discrepancy seem most likely. For instance, Mauss et al. (2005) have suggested that a poorer psychophysiological correspondence is likely in emotions that are not as strong, or in emotional states with a more dominant cognitive component (which may certainly be said of most laboratory settings). Additionally, there is evidence of social desirability playing a role in the dissociation between objective and subjective measures of stress responses (Mauss et al., 2005; Lerner et al., 2007).

Second, although the timing used in the present study is very common, as many other studies have implemented similar stress induction paradigms and observed their effects on subsequent tasks or phenomena (Payne et al., 2002; Roelofs et al., 2005; Guez et al., 2016), it does prevent us from say anything about social media use at the peak of stress. Because when momentary wellbeing was assessed and the monitoring phase took place, the stressor was already dealt with, in the sense that the presentation was already prepared and given. Participants may at that point have been experiencing a sense of relief and/or accomplishment. Many participants in our study expressed this sentiment. It is possible that bi-directional relationships between social media use and wellbeing may be found when stress is highest, which would be during the anticipatory phase of stress induction tasks. There is indeed literature indicating that for social-evaluative tasks (such as the currently used Leiden Public Speaking Task or the similar Trier Social Stress Test) stress response is highest in the period directly preceding the actual stress task (Skoluda et al., 2015).

Regardless, what the currently designed study allows us to draw conclusions about is social media directly following a stressor. Although we want to be clear that this is not what we initially set out to do, such conclusions may in fact be no less valuable (also explaining why the timing of stress effect measures is the way it is, in many studies). Many situations may be imagined in which social media are not used during but (immediately) after a stressor (e.g., stressful school exams, after a fight with a family member, after a hectic day of work), and studies discussing coping following stressful events are far from uncommon (Spurrell and McFarlane, 1993; North et al., 2001; Ducottet and Belzung, 2004; Weinstein and Ryan, 2011; Foster et al., 2012; Ruscio et al., 2015; van Ingen et al., 2016). As such, we believe the timing of the stress manipulation and social media measurement to be more than adequate in representing everyday life.

Another potential explanation for the scarcity of relationships in our study is our rather short monitoring period. The design choice of limiting the monitoring period to 10 min has been made due to participant burden and time constraints (Griffioen et al., 2020a). It is possible that a number of effects, such as an effect of stress on time spent on social media, were not found because a ceiling was hit: social media use is so popular and frequent that for such an effect to manifest a larger time frame may have been needed. This potential explanation is supported by the fact that there are people in our sample who spent all of the 10 min on social media, see Table 1. Similarly, changes in the way social media are used might still manifest at a later point in time, rather than in the 10 min immediately following the stressor. And lastly, it seems likely that qualitative aspects of social media use, such as motivation, activity and the social ties, involved only start to make a difference when they occur frequently throughout one’s pattern of use (rather than if they occur only sometimes), which is something we are not able to assess using this paradigm. Such a cumulative effect on (mental) wellbeing, although not yet demonstrated in the context of social media use, has been shown in other contexts (Mollart et al., 2009; Wallace et al., 2016).

The effect that we did find (i.e., the interaction of time spent on social media with stress level on heart rate change) is remarkable because much of the recent literature on social media use and wellbeing has suggested that it is not quantity but quality that is insightful when it comes to social media’s relationship to wellbeing. However, the quantity of social media use that we have measured here is not just regular ‘quantity’. Instead, it is likely more meaningful, since it happens directly after a stressor, which is a period for which it has been shown that cognitive and emotional processes may briefly be altered (Kohn et al., 2017; Joëls et al., 2018). It is therefore possible, that in such (sensitive) times, quantity does play a role.

In conversations with our participants, many indicated that they visited social media to see fun things and pass the time. Spending more time in such a space after a stressful event when you are already stressed to begin with might thus indeed be related to a greater calming of the nerves. Future studies will need to investigate whether this effect truly holds. Interestingly, the interaction effect did not extend to changes in subjective physiological arousal. This might have to do with the fact that subjective reporting of such physiological changes requires sufficient sensitivity. There is ample interindividual variation in interoceptive awareness (i.e., how sensitive people are to bodily changes (Murphy et al., 2019). Difficulties perceiving such (subtle) bodily changes could explain why there was an interaction with the more ‘objective’ heart rate measure but not with the subjective physiological arousal scores.

### Future Directions

Although the lack of relationships in our study is surprising when compared to previous studies, in some respects the findings presented here have to be seen on their own. Not only have we used objective data when self-report alone would fall short, we have also made sure to make the most out of self-report where it is valuable, namely, in providing context and depth to insights around social media use. We would like to stress the importance of such methods in the field of social media use and wellbeing, as such detailed combination of personal experience and objective data is unfortunately still a rarity. This first study with this paradigm has also brought forward clear suggestions for future studies in the field of social media use and wellbeing.

In order to assess social media behaviours in the context of stress regulation, we suggest future studies to match monitoring with the anticipatory stress phase. This way, stress levels remain high throughout the monitoring phase, thus allowing for assessment of actual stress-regulatory behaviours. Additionally, we think that it remains important for future studies to investigate the bi-directional relationship between wellbeing and social media use using the same degree of reliability and specificity of data as implemented here, but over a longer period of time (Reeves et al., 2019). Our study results suggest that there are no short-term effects or relationships to be found between wellbeing and social media use, and vice versa. However, it continues to be unclear whether such relationships may manifest if behaviour is measured over longer periods of time. Ecological momentary assessment methods (Van Berkel et al., 2018) in combination with passive sensing of participants’ behaviours are a likely and promising avenue for this (Lind et al., 2018), allowing for measuring behaviours over larger time frames, while preserving a higher degree of specificity and ecological validity. Many great efforts are currently made in the field to improve accuracy and quality of such data collection methods (Chang et al., 2019; Van Berkel et al., 2019).

## Conclusion

Our study has shown that acute social stress does not lead to immediate changes in social media use. Additionally, social media use following a social stressor is on most accounts *not* related to subsequent changes in momentary wellbeing, for good, nor bad. Although our conclusions can be only restricted to a short time frame, our newly developed paradigm provides opportunities to gain more reliable insights regarding young people’s social media and digital technology use.

## Data Availability Statement

The raw data supporting the conclusions of this article will be made available by the authors upon request.

## Ethics Statement

The studies involving human participants were reviewed and approved by Ethics Committee of the Faculty of Social Sciences at Radboud University Nijmegen. The patients/participants provided their written informed consent to participate in this study.

## Author Contributions

All authors contributed to conception and design of the study, manuscript revision, read and approved the submitted version. NG performed the statistical analyses and wrote the first draft of the manuscript.

## Funding

This study has been funded by the European Research Council (ERC) Consolidator Grant, grant number 683262 to IG.

## Conflict of Interest

The authors declare that the research was conducted in the absence of any commercial or financial relationships that could be construed as a potential conflict of interest.

## Publisher’s Note

All claims expressed in this article are solely those of the authors and do not necessarily represent those of their affiliated organizations, or those of the publisher, the editors and the reviewers. Any product that may be evaluated in this article, or claim that may be made by its manufacturer, is not guaranteed or endorsed by the publisher.

## Footnotes

^1^ https://osf.io/aw3mf

^2^ https://osf.io/tgvcz/
